# Implementation of an Early Mobility Initiative in a Pediatric Bone Marrow Transplant Unit

**DOI:** 10.3390/pediatric17060119

**Published:** 2025-11-05

**Authors:** Anne Swanson, Kylie James, Kimberly Fan, Akshay Sharma, Xiaomeng Yuan, Haitao Pan, Gabriela Maron, Hana Hakim, Saad Ghafoor

**Affiliations:** 1 St. Jude Children’s Research Hospital, Memphis, TN 38105, USA; kjames28@uthsc.edu (K.J.); kfan@mdanderson.org (K.F.); akshay.sharma@stjude.org (A.S.); haitao.pan@stjude.org (H.P.); gabriela.maron@stjude.org (G.M.); hana.hakim@stjude.org (H.H.); saad.ghafoor@stjude.org (S.G.); 2 College of Medicine, The University of Tennessee Health Science Center, Memphis, TN 38163, USA; 3 MD Anderson Cancer Center, Houston, TX 77001, USA

**Keywords:** pediatric, rehabilitation, mobilization, ambulation, extended-spectrum beta-lactamase, hospitalization, hematopoetic cell transplant

## Abstract

**Background/Objectives:** Children who have received hematopoietic cell transplants (HCTs) often face complex clinical courses and complications that increase their risk of functional impairments. Because of this, pediatric HCT recipients may benefit from early mobilization efforts to reduce long-term functional issues. However, early ambulation can be limited by clinical complexity and concerns about infectious transmission in HCT patients. Some patients are under contact precautions due to colonization with bacteria that produce extended-spectrum beta-lactamase (ESBL) enzymes. Our goal was to significantly increase ambulation in pediatric HCT recipients at our institution within three months of the intervention. We aimed to raise the number of ambulation events per day, the number of physical therapy (PT) visits per week, and the distance patients walked with PT per session. **Methods:** From January to October 2022, data on mobilization, demographics, and clinical characteristics were retrospectively collected from electronic health records. Starting in June 2022, we permitted ESBL-colonized patients to leave their rooms while wearing personal protective equipment (PPE), and we trained clinical staff about this in our QI initiative. **Results:** In Group 1, the ambulation rate was 1.36 times higher before the intervention than after, with an effect size of 0.3042 (*p* = 0.004 *). The ambulation rate in Group 2, admitted before the intervention, was 1.33 times higher than in Group 3, admitted after the intervention, with an effect size of 0.2856 (*p* = 0.016 *). **Conclusions:** The initiative did not increase ambulation among the targeted group. Patients ambulated more before the intervention, though these results lack statistical power. The lack of success of the intervention may be due to various factors, including the short monitoring period, retrospective data collection, difficulties with PPE use among young patients, and uncollected confounding variables related to clinical status.

## 1. Introduction

Although mortality rates for children with malignant conditions have improved in recent years, pediatric patients with oncologic conditions often experience complex clinical courses and face multiple complications during their treatment [1]. Pediatric hematopoietic cell transplant (HCT) recipients encounter additional complications that increase their morbidity, need for critical care, and resource utilization. These patients frequently have pre-existing chronic organ insufficiency, toxicity from prior treatments before HCT, intense conditioning regimens, profound immunosuppression, and unique post-HCT issues such as graft-versus-host disease (GVHD) [2]. They also undergo sedation and analgesia from various supportive care medications, are exposed to noisy inpatient environments, and need frequent nursing care, invasive interventions, or examinations. These factors can cause short- and long-term physical, cognitive, and emotional effects by disrupting sleep–wake cycles, raising the risk of delirium, impairing immunity, and leading to chronic physiological impairments [3].

Unsurprisingly, HCT recipients face a high rate of functional impairment. An analysis of childhood HCT survivors found that within 25 years of transplant, 18.8% experienced functional impairment and 28.9% faced activity limitations. The rates of functional impairment and activity limitations in HCT recipients were significantly higher than those in sibling controls and pediatric cancer patients treated without HCT. Transplants from unrelated donors, conditioning regimens with total body irradiation, and active chronic GVHD were identified as critical factors influencing risk in this population [4]. The risk of long-term functional impairments remains substantial even in HCT recipients who do not require critical care services. One study reported that pediatric HCT patients who did not need ICU admission had similar 1-year survival and functional outcomes as those who did require critical care [5].

Early rehabilitation programs are a promising way to reduce functional morbidity in critically ill patients. Studies in the adult population demonstrate that early mobilization is associated with shorter hospital stays and better functional outcomes [6]. In pediatric ICUs, early mobilization programs aimed at increasing mobilization within 72 h of ICU admission have been shown to be safe, feasible, and effective at increasing early mobilization events [7,8,9]. In 2020, we launched an early mobilization initiative called Beginning Restorative Activities Very Early (BRAVE) in our onco-critical care unit (the intensive care unit at St. Jude) to assess its safety and feasibility in the pediatric oncologic population. The early mobilization effort successfully increased mobilization within 72 h of ICU admission and lowered the percentage of patients with positive delirium screens [3]. Since pediatric HCT recipients are at high risk for developing functional morbidity regardless of critical care requirements, we hypothesize that this group will benefit from early rehabilitation initiatives.

The clinical complexity and unique needs of pediatric HCT recipients can make rehabilitation efforts more difficult to implement. HCT recipients are at high risk for colonization and infection with antibiotic-resistant organisms due to their weakened immune systems, frequent and prolonged exposures to healthcare environments, and regular antibiotic treatments [10]. Several patients on the HCT unit are under restrictive contact precautions because of colonization with antibiotic-resistant bacteria, such as bacteria producing extended-spectrum beta-lactamase (ESBL) enzymes. According to our hospital policy and CDC guidelines, HCT recipients and patients in high-risk oncologic units who are under transmission-based contact precautions should remain in their single-patient rooms [11]. These patients are permitted to leave their rooms when necessary for diagnostic and therapeutic procedures, but only when accompanied by clinical staff.

We believe that contact precautions for ESBL colonization should not prevent patients from engaging in early mobilization efforts, especially since the necessity of contact precautions in ESBL-colonized patients has been questioned. Research shows that contact precautions are associated with adverse outcomes such as falls, electrolyte imbalances, fewer interactions with healthcare staff, and higher rates of depression and anxiety. Several hospitals have found that discontinuing contact precautions in patients colonized with ESBL does not increase the risk of ESBL infection or colonization [12]. This suggests that ESBL colonization should not restrict patient mobilization to prevent the spread of ESBL-positive bacteria.

Considering their risk factors for long-term functional morbidity, we recognized the need to assess the safety and feasibility of an early mobility program in pediatric HCT recipients. We expanded our existing early mobilization protocol, BRAVE, to include HCT recipients, even those colonized with ESBL.

## 2. Materials and Methods

### 2.1. Quality Improvement Process

This Quality Improvement (QI) initiative was implemented on the Bone Marrow Transplant and Cellular Therapy Unit (Transplant Unit) at St. Jude Children’s Research Hospital, an academic, quaternary care center that treats children with hematologic and oncologic disorders. The intervention aimed to address the challenges of contact precautions for patients colonized with ESBL and to promote early mobility. Our goal was to increase Transplant Unit patient ambulation by a statistically significant amount within three months of the intervention. Specifically, we aimed to see significant increases in the number of ambulation events per day, PT visits per week, and the distance patients walked with PT per visit. The initiative was adapted from BRAVE, an early mobility initiative designed specifically for pediatric onco-critical care [3]. Before implementation, the intervention was reviewed with the Hospital Infection Control team. A formal proposal outlining personal protective equipment (PPE) protocols, environmental cleaning, and monitoring for ESBL transmission was submitted and approved, ensuring compliance with institutional safety standards.

In June 2022, we conducted educational sessions for Transplant Unit staff on the rationale and process of early mobilization in HCT recipients. The training emphasized how early mobilization helps reduce delirium and long-term functional impairment, and explained the new procedure for safely ambulating ESBL-positive patients. We reviewed the types of early mobilization activities used with our ICU patients, including both in-bed activities (passive and active range-of-motion, sitting at the edge of the bed, and active or passive repositioning) and out-of-bed activities (sit-to-stand, use of a mobility device, transferring from bed to chair, or ambulation) [3]. We recommended that staff encourage patient ambulation outside their rooms at least once per nursing shift. When assisting ESBL-colonized patients outside their rooms, the staff member present (nurse, physical therapist, or patient care assistant) was instructed to wear contact precaution PPE (gown and gloves) in accordance with hospital policy, and to help the patient don a clean gown and gloves. We provided detailed procedures for putting on and removing PPE during patient ambulation.

To conclude the educational curriculum, we highlighted the importance of patient safety and adherence to infection control measures. Bedside nurses or physical therapists were to initiate ambulation based on the patient’s clinical stability, and all ambulation sessions for ESBL-colonized patients were to be directly supervised by clinical staff to ensure safety, PPE use, and infection control compliance. To reduce the risk of transmission to other vulnerable patients, routes for ambulation were designed to avoid direct contact with others. During ambulation, other immunocompromised patients were encouraged to remain in their rooms. All mobility aids (walkers, IV pole handles) and high-touch surfaces (door handles) contacted during ambulation were to be disinfected by the accompanying staff member with hospital-grade disinfectant wipes immediately after the patient’s return to their room. Informational flyers summarizing the new policy and ambulation procedures were posted in high-traffic areas throughout the unit, such as nursing stations, break rooms, and outside patient rooms.

As a result of providing this training to various clinical disciplines, a multidisciplinary team helped improve patient mobility. PT conducted scheduled assessments and delivered structured mobilization sessions, using mobility aids (walkers, gait belts) as needed. Child Life Specialists employed developmental and motivational strategies (e.g., play activities, games, music) to reduce anxiety and promote participation in walking. Nurses and trained family members assisted with simple ambulation activities between formal PT sessions, following PPE and supervision guidelines.

### 2.2. Staff Education Process

Initial training in June 2022 included mandatory 30 min in-person sessions held at a central staff meeting location. An online tutorial covering the same material was available for staff who could not attend in person. Brief, 10 min “refresher” sessions occurred every other week during staff huddles to reinforce key points and address new concerns. Attendance was recorded for all in-person sessions. Completing the online tutorial was mandatory and tracked through the hospital’s learning management system. A multiple-choice question test was given at the end of both the in-person session and the online tutorial. A perfect score of 100% was required, and remediation was provided for incorrect answers.

Staff compliance with the education program served as our process measure, tracked through attendance and completion rates for the online module and test. A positive reinforcement system was put in place, in which unit leadership awarded small tokens of appreciation (such as public recognition) to staff members consistently observed encouraging or facilitating patient ambulation.

### 2.3. Data Collection and Analysis

We conducted a retrospective review of medical records for all admissions to the Transplant Unit from January to October 2022, covering the periods before and after the intervention, with a dedicated month in June for implementation and education. January to May 2022 was designated as the pre-intervention period, while July to October 2022 was the post-intervention period. We collected data on mobilization, demographics, and clinical characteristics, including diagnosis and transplant type. Our outcome measures included the number of ambulation events per day, the number of physical therapy sessions per week, and the distance walked (in feet) per physical therapy (PT) session. For each patient, we reviewed nursing flowsheets from the study period and recorded each mobilization event documented by nursing. Additionally, we examined documentation from each physical therapy session during each patient’s admission, noting the number of PT sessions per week and the distance each patient ambulated during therapy.

The primary measure for balance was the rate of hospital-acquired ESBL colonization, tracked through rectal surveillance culture (RSC) results for all patients before and after the intervention. We collected the existing RSC results for all patients admitted to the Transplant Unit during the study period. Our aim was to use this combined dataset to detect increased transmission of ESBL-producing bacteria associated with the intervention. An increase might suggest that ESBL-colonized patients were spreading the bacteria as they moved around outside their rooms. The unit’s overall infection rate with multidrug-resistant organisms was also monitored by Infection Control to detect changes in microbial populations.

### 2.4. Statistical Analysis

The study included three patient groups: Group 1 comprised patients hospitalized both before and after the early mobilization intervention; Group 2 comprised patients hospitalized before the intervention; and Group 3 comprised patients hospitalized after the intervention. For demographic and baseline clinical characteristics, summary statistics (mean, standard deviation, median, and range for continuous variables; frequencies and percentages for categorical variables) are reported for each group.

Comparisons of demographic and clinical characteristics between Groups 2 and 3 were made using the exact Pearson chi-square test for categorical variables and the Wilcoxon rank-sum test for continuous variables. For patients in Group 1, ambulation outcomes before and after the intervention were compared within the same individuals to evaluate changes over time.

Ambulation outcomes included the number of ambulation events per day, PT visits per week, and feet walked per PT visit. These outcomes were analyzed using Poisson regression models to estimate rate ratios that quantify the intervention’s effect. A significance level of *p* = 0.05 was used for all statistical tests. Analyses were conducted using SAS software, version 9.4, and R software, version 4.4.0. For the number of ambulation events per day and PT visits per week, both outcomes were analyzed using regression models selected to best match the distributional characteristics of each variable. Candidate models included Poisson regression, generalized linear models, and negative binomial regression, with and without zero-inflated components. Final model selection was guided by the Akaike Information Criterion (AIC) to ensure the best fit and proper handling of dispersion and zero inflation.

For Group 1, the impact of time on the ambulation rate was assessed using a Poisson regression model, and the effect of time on the PT visit rate was analyzed with a generalized Poisson regression model. For feet walked per visit, a semi-continuous variable that may include zero values, Tweedie regression models were used, both with and without zero inflation. For Groups 2 and 3, we evaluated the effect of group on the ambulation rate, defined as the total number of ambulation events per patient-day, using a negative binomial model. The impact of group (Group 2 vs. Group 3) on the PT visit rate, defined as the total number of PT visits per patient-week, was assessed using a negative binomial regression model with a zero-inflated component. Lastly, the effect of group (Group 2 vs. Group 3) on the number of feet walked with PT per visit was examined using a Tweedie regression model.

## 3. Results

### 3.1. Demographics

Eighty-five patients participated in the study: 11 were admitted both before and after the intervention (Group 1), 39 were admitted only before the intervention (Group 2), and 35 were admitted only after the intervention (Group 3). Patient characteristics for the groups are summarized in Table 1. In each group, approximately half of the patients had leukemia or lymphoma, while the rest were diagnosed with either solid tumors or non-malignant disorders. Over 50% of patients in each group received allogenic transplants. No statistically significant differences in demographic variables were observed between patients admitted before and after the intervention.

### 3.2. Ambulation as an Outcome Measure

Ambulation rate was defined as the total number of ambulation events per patient day. Patients admitted both before and after the intervention (Group 1) had a higher ambulation rate prior to the intervention (*p* = 0.0015). Similarly, these patients walked longer distances with PT before the intervention compared to after (*p* < 0.0001). (Table 2). Patients whose hospital stays occurred only before the intervention (Group 2) had a higher ambulation rate than those admitted after the intervention (Group 3) (*p* < 0.0001). Group 2 also ambulated longer distances with PT than Group 3 (*p* < 0.0001). There was no statistically significant difference in the total number of PT visits between the groups (Table 3). However, after calculating dispersion and corresponding *p*-values for these Poisson models, the results showed significant overdispersion for all rate ratios that were statistically significant.

The results of the linear regression model for Group 1 are presented in Table 4. In Group 1, the ambulation rate was 1.36 times higher before the intervention than after (*p* = 0.004). The total number of PT visits per patient-week was 3.28 times higher before the intervention than after, and the number of feet walked per PT visit was 2.32 times higher before the intervention than after. However, neither of these findings was statistically significant.

The results of the linear regression model for Groups 2 versus 3 are shown in Table 5. After evaluating the effect of group on the ambulation rate, our findings indicated that the ambulation rate in Group 2, which was admitted before the intervention, was 1.33 times higher than in Group 3, admitted after the intervention (*p* = 0.016). The PT visit rate in Group 2 was 1.37 times that of Group 3, and the feet walked per visit in Group 2 was 1.45 times that in Group 3, although neither difference was statistically significant.

### 3.3. ESBL Colonization

Out of 186 admissions to the transplant unit from January to May 2022, RSCs were collected from 85 patients. After the intervention, from July to September 2022, RSCs were collected from 59 of the 154 admitted patients. In the three months before the intervention, three previously uncolonized HCT recipients acquired ESBL colonization, compared to four patients who acquired new ESBL colonization after the intervention (Table 6).

There was no clear evidence that the intervention was associated with a higher rate of ESBL colonization, although this data should be interpreted carefully. The decision to collect RSCs depends on various clinical criteria, and many Transplant Unit patients did not have RSCs collected, so surveillance culture coverage was limited.

## 4. Discussion

This early mobilization intervention did not increase the number of ambulation events per day, PT visits per week, or distance walked during PT within three months after the intervention. In fact, patients in all three groups had higher ambulation rates before the intervention than after. This was the only comparison that produced a statistically significant result that was not overdispersed. Nonetheless, there was no clinically meaningful increase in the number of patients colonized with ESBL following the intervention. Even if the intervention had been successful, we would not expect a significant rise in ESBL infections or colonization, as patients wear PPE outside their rooms. The lack of success with the intervention may be due to contextual barriers, the patient population, the implementation process, or data collection issues.

### 4.1. Contextual and Patient-Specific Barriers to Success

Contextual factors, such as psychological and logistical barriers, may have discouraged ambulation after the intervention. Patient mobilization is often supported and encouraged by their families, many of whom have more time to visit during the summer months before the intervention takes place. The post-intervention period happened in August through September, when the cold and flu season is quickly approaching. It is possible that, despite education on the importance of mobilization, staff and patient families may have unintentionally encouraged less ambulation outside of rooms during these months to protect immunocompromised patients. Additionally, wearing PPE can be challenging for some children and may prevent them from leaving their rooms to ambulate. Future research should focus on evaluating the need for contact precautions in pediatric patients colonized with ESBL.

Additionally, factors related to the patient population may have influenced the intervention’s success. Some patients’ clinical conditions might have worsened over time, leading to reduced ambulation. The clinical complexity of pediatric HCT recipients likely played a major role in their ability to walk. These patients often face multiple complications, including severe mucositis, nausea, vomiting, and the need for various parenteral medications, which are often sedating. They may also experience acute or chronic organ insufficiency, which can limit their participation in mobilization activities. Moreover, the frequent use of sedation and pain management, along with continuous environmental noise and invasive procedures, may further hinder their ability for early ambulation.

### 4.2. Barriers to Success Related to Study Design, Implementation, and Analysis

Several factors related to study design and implementation may have contributed to the lack of the desired outcome. The intervention was limited to printed posters and staff education, which likely was insufficient to induce a change in mobility among a complex pediatric patient population. Additionally, the intervention period might have been too brief to observe significant shifts in ambulation behavior. The three-month post-intervention period may not have been sufficient for patients, families, and staff to fully adapt to the new mobilization guidelines.

Another design factor that may have affected ambulation is the presence of unmeasured clinical variables. Variables such as the specific conditioning regimen, the presence and severity of mucositis, GVHD status, the use of sedation or pain medication, and the burden of central or peripheral lines and other external medical devices are potential confounders that were not examined in our study. Future research should focus on collecting and analyzing these variables to better understand their impact on mobilization outcomes.

Finally, factors related to data collection and analysis may have influenced the intervention’s success. Our data were gathered retrospectively and were limited to what could be retrieved from medical records. As a result, we could not evaluate the accuracy of nursing documentation or the consistency of documentation among different nurses on the unit. Consequently, ambulation may have been underreported. A large part of the statistical analyses relied on Poisson regression models for ambulation outcomes. Given the relatively small sample size and presence of overdispersion of count data, these results should be viewed with caution. Even after addressing overdispersion by utilizing a negative binomial regression, generalizability and statistical power remain low.

### 4.3. Recommendations for Future Studies

Clinicians can use various strategies to effectively implement mobilization initiatives in pediatric HCT recipients. A multidisciplinary approach is particularly valuable, as involving physicians, physical and occupational therapists, nursing staff, and child life specialists increases the chances of promoting ambulation in this group. Incorporating personalized rehab plans and providing ongoing education for staff and families may improve the feasibility and success of early mobilization programs. Utilizing serial Plan-Do-Study-Act (PDSA) cycles [13] to assess early mobilization efforts in pediatric cancer patients can help clinicians better identify barriers to movement in this population and allow for longer study periods, encouraging improvements in intervention design. As we continue to perform PDSA cycles for this QI project, we will implement and assess a standardized documentation process for patient mobilization and begin documenting a number of clinical variables, including central and peripheral line burden, sedating medications, and the presence of nausea or vomiting. Larger studies that perform statistical models such as negative binomial regression may generate generalizable results with more statistical power. Collecting prospective data and applying tailored analytic strategies will further enhance accuracy and reproducibility in future research.

## 5. Conclusions

Although increasing ambulation was not successful, our study emphasizes the importance of addressing the specific needs and challenges faced by pediatric HCT recipients. Early mobilization remains a promising strategy to reduce long-term functional problems, and additional efforts are necessary to customize these approaches for HCT patients. Future QI projects with similar goals will benefit from a comprehensive, multidisciplinary education program with multiple interactive sessions, a monitoring period longer than 3 months, prospective data collection, and the inclusion of variables related to illness severity. Identifying and overcoming barriers to mobilization in this complex patient population is crucial for improving long-term functional outcomes and overall quality of life.

## Figures and Tables

**Table 1:** Demographic and baseline characteristic variables.

Variables	Group 1 Summary Statistics (N = 11)	Group 2 (N = 39)	Group 3 (N = 35)	Group 2 and 3 Combined (N = 74)	Group 2 and 3 Comparison *p*-Value **
Age at admission (years)					
Mean (SD)	9.8 (4.4)	13.1 (5.9)	13.7 (6.0)	13.4 (5.9)	0.6650
Median (Min, Max)	8.5 (4.4, 16.3)	13.3 (2.9, 25.2)	14.0 (3.4, 24.2)	13.6 (2.9, 25.2)	
Gender					
Female	4 (36.4)	21 (54)	16 (46)	37 (50)	0.6418
Male	7 (63.6)	18 (46)	19 (54)	37 (50)	
Race					
Black/African American	3 (27.3)	11 (28)	9 (26)	20 (27)	0.5138
Caucasian	7 (63.6)	26 (67)	25 (71)	51 (69)	
American Indian/Alaskan Native		0 (0)	1 (3)	1 (1)	
Asian/Pacific Islanders		2 (5)	0 (0)	2 (3)	
Other	1 (9.1)				
Ethnicity					
Hispanic/Latino	3 (27.3)	7 (18)	6 (17)	13 (18)	1.0000
Non-Hispanic/Latino	8 (72.7)	30 (77)	29 (83)	59 (80)	
Unknown		2 (5)	0 (0)	2 (3)	
Oncology diagnosis before transplant					
Leukemia/Lymphoma	7 (63.6)	20 (51)	16 (46)	36 (49)	0.9086
Non-malignant disorder	2 (18.2)	12 (31)	12 (34)	24 (32)	
Solid Tumor	2 (18.2)	7 (18)	7 (20)	14 (19)	
Transplant category					
Allogenic HCT	7 (63.6)	23 (59)	23 (66)	46 (62)	0.5649
HAPLO	6 (54.5)	12 (31)	15 (43)	27 (36)	
MSD	1 (9.1)	4 (10)	3 (9)	7 (9)	
MUD		7 (18)	5 (14)	12 (16)	
Autologous HCT	1 (9.1)	11 (28)	6 (17)	17 (23)	
Immune Effector Cell Therapy	3 (27.3)	5 (13)	6 (17)	11 (15)	
Number of days between admission and transplant					
Mean (SD)	−131.3 (324)	−164.3 (409.5)	−6.5 (77.2)	−89.7 (310.4)	0.0515
Median (Min, Max)	9 (−931, 82)	7.0 (−1729.0, 73.0)	9.0 (−329.0, 53.0)	8.0 (−1729.0, 73.0)	
Total transplant number					
Mean (SD)	1.9 (1.4)	1.3 (0.5)	1.2 (0.4)	1.3 (0.5)	0.1650
Median (Min, Max)	1.0 (1, 5)	1.0 (1.0, 3.0)	1.0 (1.0, 2.0)	1.0 (1.0, 3.0)	
Length of hospitalization (days)					
Mean (SD)	33.2 (22.9)	26.2 (20.5)	25.3 (16.2)	25.8 (18.5)	0.9482
Median (Min, Max)	29.9 (1.1, 85.5)	23.4 (0.4, 93.1)	27.2 (1.9, 78.4)	25.3 (0.4, 93.1)	
Patient-days pre-intervention					
Mean (SD)	21.8 (14.9)				
Median (Min, Max)	21.6 (0.5, 49.8)				
Patient-days post-intervention					
Mean (SD)	11.4 (12.5)				
Median (Min, Max)	6.1 (0.5, 44.7)				

For nominal variables, the ‘summary stats’ are the frequency counts (%); for numeric variables, the ‘summary stats’ are the corresponding statistics of the row name. ** For group comparisons, the *p*-value of nominal variables was derived from the exact Pearson chi-square test; the *p*-value of numeric variables was derived from the Wilcoxon rank-sum test. For nominal variables, the numbers are the frequency counts (%); for numeric variables, the numbers are the corresponding statistics of the row name. SD = standard deviation. HAPLO = haploidentical donor. MSD = matched sibling donor. MUD = matched unrelated donor.

**Table 2:** Ambulation outcomes, Group 1.

Variables	1-Pre (N = 11)	2-Post (N = 11)	Estimate of Time Effect	Rate Ratio	*p*-Value	Dispersion (*p*-Value)
Ambulation events per day						
Mean (SD)	1.6 (1.2)	1.4 (1.5)	0.3158	1.37	0.0015 *	14.79 (<1 × 10^−6^ *)
Median (Min, Max)	1.4 (0.0, 4.0)	0.9 (0.0, 5.4)				
PT visits per week						
Mean (SD)	0.5 (0.4)	0.2 (0.6)	1.1962	3.31	0.0542	1.28 (0.356)
Median (Min, Max)	0.5 (0.0, 1.2)	0.0 (0.0, 2.1)				
Feet walked per visit						
Mean (SD)	595.8 (432.6)	250.0 (70.7)	0.8460	2.33	<0.0001 *	572 (<1 × 10^−6^ *)
Median (Min, Max)	625.0 (0.0, 1250.0)	250.0 (200.0, 300.0)				

SD = standard deviation; * statistically significant.

**Table 3:** Ambulation outcomes, Group 2 vs. Group 3.

Variables	Group 2 (N = 39)	Group 3 (N = 35)	Estimate of Group Effect	Rate Ratio	*p*-Value	Dispersion (*p*-Value)
Ambulation events per day						
Mean (SD)	1.2 (0.7)	1.0 (0.6)	0.2730	1.31	<0.0001 *	6.78 (<1 × 10^−6^ *)
Median (Min, Max)	1.2 (0.0, 2.6)	1.0 (0.0, 2.8)				
PT visits per week						
Mean (SD)	0.6 (1.5)	0.5 (0.5)	−0.1413	0.87	0.3931	2.17 (<1 × 10^−6^ *)
Median (Min, Max)	0.0 (0.0, 9.1)	0.4 (0.0, 1.7)				
Feet walked per visit						
Mean (SD)	819.8 (458.5)	600.2 (477.6)	0.4134	1.51	<0.0001 *	1184.42 (<1 × 10^−6^ *)
Median (Min, Max)	724.0 (100.0, 1629.0)	500.0 (0.0, 1500.0)				

SD = standard deviation; * statistically significant.

**Table 4:** Generalized linear regression results for Group 1.

Variables	Estimate of Time Effect (*p*-Value)	Rate Ratio	*p*-Value of Model Diagnosis **	Dispersion (*p*-Value)
Ambulation events per day	0.3042 (0.004 *)	1.36	0.95	1.67 (0.16)
PT visits per week	1.1872 (0.053)	3.28	0.87	1.39 (0.23)
Feet walked per visit	0.8437 (0.303)	2.32	0.59	1.79 (0.26)

* Statistically significant. ** *p*-value is calculated using the Kolmogorov–Smirnov test between simulated residuals and uniform distributions.

**Table 5:** Generalized linear regression results for Group 2 vs. Group 3.

Variables	Estimate of Group Effect (*p*-Value)	Rate Ratio	*p*-Value of Model Diagnosis **	Dispersion (*p*-Value)
Ambulation events per day	0.2856 (0.016 *)	1.33	0.16	0.75 (0.12)
PT visits per week	0.3184 (0.153)	1.37	0.67	1.16 (0.33)
Feet walked per visit	0.3716 (0.098)	1.45	0.88	1.08 (0.69)

* Statistically significant. ** *p*-value is calculated using the Kolmogorov–Smirnov test between simulated residuals and uniform distributions.

**Table 6:** ESBL colonization in HCT recipients.

	Before Intervention (January Through May 2022)	After Intervention (July Through September 2022)
Number of HCT recipients with RSCs collected	85	59
Number of ESBL (−) RSCs (*Rate*)	79 (*0.929*)	44 (*0.746*)
Number of ESBL (+) RSCs (*Rate*)	6 (*0.071*)	15 (*0.254*)
Number of previously uncolonized ESBL (+) RSCs (*Rate*)	3 (*0.035*)	4 (*0.068*)

ESBL = extended-spectrum beta-lactamase. HCT = hematopoietic cell transplant. RSC = rectal surveillance culture.

## Data Availability

The datasets presented in this article are not readily accessible because they contain patient-protected health information (PHI).

## Abbreviations

The following abbreviations are used in this manuscript:

BRAVE	Beginning Restorative Activities Very Early
CDC	Centers for Disease Control and Prevention
ESBL	Extended-Spectrum Beta-Lactamase
GVHD	Graft-versus-host-disease
HCT	Hematopoietic Cell Transplant
ICU	Intensive Care Unit
PT	Physical Therapy
PDSA	Plan, Do, Study, Act
RSC	Rectal Surveillance Culture
QI	Quality Improvement
